# Temporal Recall Strategies in Rheumatoid Arthritis Patients’ Emotionally Intense Life Events

**DOI:** 10.3390/ijerph21060759

**Published:** 2024-06-11

**Authors:** Fanni Balikó, Krisztina Csókási, Melinda Pohárnok, Orsolya Vincze, Gábor Kumánovics, Marcell Deme, Preston Alexander Long, Tanja Stamm

**Affiliations:** 1 Institute of Psychology, University of Pécs, 7621 Pécs, Hungary; baliko.fanni@pte.hu (F.B.); csokasi.krisztina@pte.hu (K.C.); poharnok.melinda@pte.hu (M.P.);; 2 Department of Rheumatology and Immunology, University of Pécs, 7621 Pécs, Hungary; kumanovics.gabor@pte.hu (G.K.); deme.marcell@pte.hu (M.D.); 3 Department of Outcomes Research, Medical University of Vienna, 1090 Vienna, Austria; preston.long@meduniwien.ac.at

**Keywords:** adaptation, life story, rheumatoid arthritis, recalling patterns, patient-reported outcome, savoring

## Abstract

Background: Rheumatoid arthritis (RA) patients often encounter psychological challenges due to chronic pain, fatigue, side effects of medications, and disability. This study examines the relationship between autobiographical narratives and recollection patterns in RA patients. We investigated how different recall strategies for positive life events affect the emotional processing of negative episodes. We hypothesized that vividly recalling positive life events provides psychological resources that support a more intense emotional elaboration of stressful memories, allowing individuals to delve deeper into negative life experiences. Additionally, we explored the impact of these perspectives on self-reported well-being and physical health, proposing that re-living positive events improves overall well-being. Methods: We collected and analyzed high-point and low-point life-story episodes from 60 RA patients (85% female; age mean 61 ± 11 years; range 37–79) using episodic narrative interviews and the Narrative Categorical Content Analysis algorithm (NarrCat). Participants were categorized into 2 clusters based on their temporal perspective during high-point episodes: 25 used a Retrospective viewpoint, while 35 employed a Re-experiencing strategy. Depression and anxiety were assessed with the Hospital Anxiety and Depression Scale (HADS), and functioning was measured using the Health Assessment Questionnaire (HAQ). Results: The Re-experiencing group, which was more likely to articulate their high-point episode in vivid and real-time narrative, used more psychological perspectives (U(58) = 223, *p* < 0.01) and showed heightened emotional frequency (U(58) = 280, *p* < 0.05; positive: U(58) = 328, *p* < 0.05; negative: U(58) = 278, *p* < 0.05) in low-point episodes. No significant difference emerged between the two groups regarding psychological state (anxiety, depressive symptoms) and physical impairment. Conclusions: Vividly recalling positive events may facilitate a deeper exploration of negative memories. The Re-experiencing group showed increased positive emotions during low points, suggesting better emotion regulation. However, no significant association was found between recalling strategies, psychological state, and physical impairment. This indicates that further research is needed to determine whether re-experiencing positive life events is adaptive or maladaptive.

## 1. Introduction

People exhibit considerable psychological diversity, and one of the key domains where this variability becomes apparent is in how they recall their life stories [1,2]. Inflammatory conditions could impact both physical and mental well-being, leading to heightened fatigue and a predominance of negative emotions. Individuals with RA exhibit a higher likelihood of experiencing anxiety and are more prone to developing depressive symptoms compared to the general population [3]. This could raise the question whether living with severe physical impairment and higher vulnerability of negative feelings and mood disorders influences the recalling strategies of positive and negative life events of the patients.

Researchers have recently shifted their focus to explore the connection between valanced memory and emotion regulation, examining the specific strategies individuals utilize to navigate emotions once they are evoked [4]. The life-story narrative represents an individual’s internalized and dynamic portrayal of the self, merging the reconstructed past with an envisioned future [2]. Within the life-story narrative, key moments, notably high- and low-point episodes, serve as key junctures. These emotionally charged episodes in the narrative frequently unveil essential personality themes that are interconnected with emotional processes and psychological well-being [5].

Past research with healthy individuals has delved into the analysis of emotion regulation, highlighting the pivotal role played by high- and low-point autobiographical episodes, although less is known about the memory characteristics of RA patients. Positive emotion regulation strategies involve adaptively responding to negative emotional experiences, such as focusing on positives, while negative strategies amplify or prolong negative emotions through maladaptive responses like catastrophizing and rumination [4]. Adaptive emotion regulation strategies result in adequate responses to negative emotions, including reframing negatives and focusing on positives. On the contrary, maladaptive emotion regulation strategies indicate less adequate responses, amplifying or prolonging negative emotions through actions like catastrophizing and rumination [5]. Most of the previous studies [5,6,7] explored the regulation of negative emotions, as their dysregulation might have more detrimental effects. However, the regulation of positive emotions could also be influential in adaptive functioning and mental well-being. In line with this, Quoidbach and colleagues [8] explored how different memory strategies impact mental well-being, focusing on savoring and dampening strategies of positive emotions in a non-clinical sample. Analyzing the levels of general happiness, positive affect, and dispositional positive emotion regulation strategies of 282 participants, they found that strategies, such as concentrating on the present moment, positive rumination or vividly recalling positive memories increased positive affect and enhanced life satisfaction. Conversely, distraction lessened the positive affect, and negative rumination reduced life satisfaction during positive events. Savoring refers to the ability to consciously amplify and prolong the pleasure derived from positive experiences, and it is mostly studied in positive emotion regulation research [8,9]. Its relevance extends beyond the general population to clinical contexts, including studies with cancer patients [10,11]. The importance of savoring in complex therapies for cancer has also been shown, with a higher ability to savor positive experiences predicting greater happiness and higher life satisfaction [11]. Hou and colleagues [10] studied 263 patients with different types of cancer, and their results showed that in individuals who reported high levels of savoring, no significant association emerged between the physical symptoms caused by the disease and the development of depressive symptoms. Considering the similarities between the challenges faced by cancer patients and RA patients, such as dealing with loss and adapting to ongoing changes, savoring could be a helpful coping strategy. In the health context, asking about a patient’s pleasure can be a positive intervention, granting permission to delve beyond the illness. The incorporation of positive emotions into clinical practice empowers individuals to present the positive aspects of their lives [12]. Different approaches have been identified to prolong and increase positive emotional experiences, of which we pay particular attention to Positive Mental Time Travel by vividly remembering or anticipating positive events [8].

Temporal perspectives of narratives can be treated as anchors to locations, from which the information is recalled. Narrators’ subtle shifts in perspective when recalling significant life events may communicate hidden information about their degree of adjustment. In instances of a Retrospective viewpoint, the narrator’s spatial and thematic positions diverge: the narrator’s spatial orientation is rooted in the present narrative, while the life story content is situated in the historical, past context of narrated events. In contrast, adopting a Re-experiencing perspective aligns the spatial coordinates of the narrator’s position and the life story content, as the narrator transports the narrative from the past of the events to the present of the narration [13]. Consequently, both the narrator’s spatial orientation and thematic content coincide in the current narrative (Table 1.)

Research on threatened identities and emotion regulation investigated the psychological significance of these perspectives [13]. Outcomes revealed that employing the Retrospective form is linked to elaboration progress, emotional detachment, and a sense of conclusion. Contrastingly, the Re-experiencing form (the vivid re-living of a negative past episode), accompanied by grammatical structures suggesting ongoing and unresolved issues, implies a more problematic identity state and challenges in coping mechanisms, as observed in studies that investigated identity-relevant biographic episodes of vulnerable populations—gay men and women experiencing unsuccessful in vitro fertilization [13]. The Positive Mental Time Travel strategy identified by Quoidbach and colleagues [8] has common characteristics with the Re-experiencing time perspective as both include vivid recall. However, the use of the Re-experiencing strategy can be maladaptive or adaptive, depending on the valance of the emotional event.

Rheumatoid arthritis (RA) is a persistent autoimmune condition marked by joint inflammation, fatigue, chronic pain, and stiffness [3]. The disease’s course is often unpredictable, and fluctuating symptoms keep patients in a chronic state of alert [14]. RA is more prevalent in women, with an overall gender ratio of approximately 3:1 [15]. The prevalence increases with age, peaking in midlife, possibly explaining the higher frequency of diagnoses between the ages of 56 and 58 [16]. The psychosocial impact of RA encompasses adverse changes in both professional and personal spheres, leading to isolation, deprivation, and the loss of meaningful activities, ultimately affecting psychological well-being. Consequently, clinically significant depressive and anxiety symptoms commonly afflict RA patients, who may encounter challenges in experiencing joy and pleasure due to the loss experience that accompanied their unfavorable symptoms [3,14]. The exploration of memories associated with both positive and negative life episodes gains significance when reflecting on their life experiences. Research consistently shows that daily emotional states and distress can impact RA symptoms [7].

Focusing on the positive aspects of patients’ lives might emerge as a beneficial intervention strategy. RA patients are frequently confronted with negatively phrased questions about their pain, disability, and fatigue. However, by focusing their attention on positive memories and emotions, it is possible to focus their attention on the positive aspects [6,11,17]. Frederickson’s broaden-and-build theory posits that the higher levels of positive affect lead to greater capacity of psychological and physiological recovery from stressful events [9]. Positive emotions broaden mindsets, expand the range of cognitions and behaviors to build an individual’s physical, intellectual, and social resources, and may improve health and pain severity. Studies indicate that the presence of positive emotions diminishes the stressful impact of chronic pain, particularly observed in patients with rheumatoid arthritis [18].

The way memories are recalled is intricately connected to the strategies individuals employ to regulate their emotions, which can significantly contribute to maintaining well-being in RA [14]. Therefore, the current study examines the associations between memories of emotionally intense life events, self-report indices of mental well-being and subjective evaluations of physical impairment.

We hypothesized an association between the temporal perspective and the narrative characteristics of emotionally intense life events. More precisely we presumed an association between the temporal perspective adopted during high-point episodes and the narrative characteristics of low-point episodes. High points were characterized by moments of profound positivity, such as happiness, joy, or a sense of excitement within their narrative. On the other hand, low points represented adverse life experiences, marked by distress or challenges [2]. Specifically, we anticipated differences in the emotional and psychological elaboration of low-point episodes between individuals utilizing a Retrospective perspective during high-point episodes and those employing a Re-experiencing perspective during such episodes. Our underlying assumption, based on previous considerations within the context of the broaden-and-build theory, is that vivid positive emotions enhance resilience to distress and aid in physiological recovery from negative emotional arousal. Additionally, deeper immersion in positive emotions may help build psychological resources essential for effectively coping with emotionally intense life events [9]. Therefore, individuals capable of immersing themselves in and re-living positive emotional experiences may engage in a more detailed emotional and psychological elaboration of negative emotions. Secondly, based on Quoidbach and colleagues’ [8] findings, we investigated the potential links between temporal perspectives of high-point episodes, self-report indices of mental well-being and subjective evaluations of physical impairment. We assumed that the ability of re-experiencing positive events in the present moment might increase the well-being of individuals.

## 2. Materials and Methods

### 2.1. Sample

The study comprised 60 Hungarian rheumatoid arthritis patients (51 female [85%]; age mean 61 ± 11 years, are range: 37–79). In terms of marital status, most of the participants were married or in a long-term relationship. The sample consisted mainly of people living with the disease long term (>10 years). Almost 80% of the sample were retired or on disability pension. Further demographic characteristics of the sample are presented in Table 2 (see below in Section 3.1). The participants in this study were hospitalized individuals receiving treatment at the Rheumatology and Immunology Department of the Clinical Centre of Pécs, Hungary. Inclusion criteria comprised a seropositive RA diagnosis and active participation in biological therapy. Exclusion criteria involved comorbidity with another autoimmune disease or other chronic illness (e.g., tumor, oxygen-demanding COPD, severe heart failure). This approach enabled the investigation to discern the psychological characteristics (e.g., recalling strategies) of RA patients independent of other somatic conditions. The study subjects did not receive financial compensation for participating in the study.

### 2.2. Procedure

Following the agreement to participate, patients were directed to a designated room after the hospital visit. In this setting, they provided written consent. With a trained interviewer, they underwent the semi-structured interviews based on McAdams’ Life Story Interview [1,2], exploring high- and low-point episodes. Life stories are valuable for exploring the various themes, needs, and life goals of the narrator, encompassing temporal perspectives that involve actively re-living emotions, possibly feeling overwhelmed, or experiencing emotional closure [2]. High points were defined as notably positive experiences, marked by happiness, joy, excitement, or an overall sense of wonder within the narrative. Conversely, low points represented the antithesis of the high point, like stressful and challenging life experiences. Participants were prompted to reflect on their entire lives and pinpoint scenes that stood out as high and low points and describe them as detailed as possible. Later, participants completed questionnaires aiming to assess their depression and anxiety symptoms (Hospital Anxiety and Depression Scale) and physical functioning (Health Assessment Questionnaire), in alignment with the research focus. Interviews were conducted with consent, anonymized using patient codes, and approved by the Hungarian Medical Research Council (protocol code BM/15367-1/2023). The used methods and data processing were in accordance with the Helsinki Declaration.

### 2.3. Measures

In evaluating the psychological and physical characteristics of our sample, we employed the Hospital Anxiety and Depression Scale (HADS) [19] and Health Assessment Questionnaire (HAQ) [20] to measure anxiety, depression, and physical functioning.

The Hospital Anxiety and Depression Scale (HADS) comprises fourteen items divided into two subscales, each focusing on anxiety and depression with seven items. Scoring for each item ranges from zero to three, and the questionnaire provides a separate score for each dimension. HADS anxiety and depression scores ≥ 8 have been reported as cut-offs indicating elevated symptoms (possible/subclinical cases), and scores ≥ 11 indicate the presence of clinically relevant anxiety or depressive symptoms (definite cases) [19]. The Cronbach’s alpha coefficient yielded a value of 0.78 for the anxiety sub-scale and 0.86 for the depression sub-scale, indicating the reliability of the questionnaire.

The Health Assessment Questionnaire (HAQ) is a self-administered tool used to assess disability. It is one of the earliest patient-reported outcome (PRO) instruments designed to capture patient-centered outcomes [20]. The HAQ is based on five dimensions: disability, pain, medication effects, costs of care, and mortality. Consisting of 20 questions across 8 categories, respondents rate their difficulty level on a scale from 0 (no difficulty) to 3 (unable to perform), with the highest score within each category representing the overall score of the category. HAQ scores of 0 to 1 are generally considered to represent mild to moderate difficulty, scores of 1 to 2 represent moderate to severe disability, and scores of 2 to 3 represent severe to very severe disability [20]. The internal consistency of the HAQ, evaluated using Cronbach’s alpha, is reported as 0.94.

### 2.4. Data Analysis

#### 2.4.1. Interview Data

**Figure 1:**
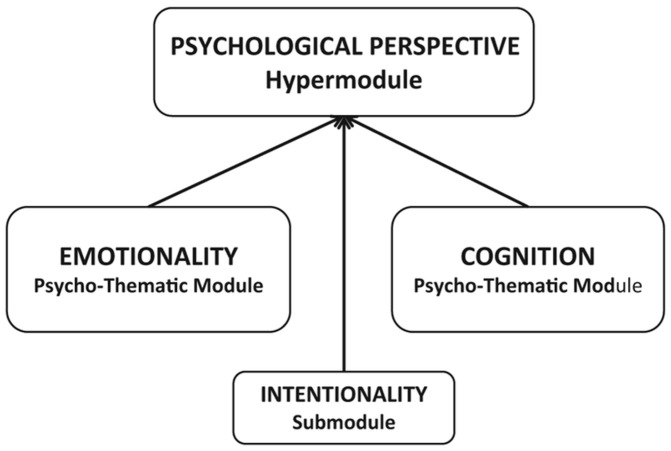
Submodules of the Psychological Perspective hypermodule of NarrCat (adapted from the original work of László et al. [21]).

After transcribing the interviews into verbatim text, each high- and low-point episode underwent quantitative analysis using the Narrative Categorical Content Analysis algorithm (NarrCat). NarrCat is a toolkit using six Psycho-Thematic Modules that quantifies psychological processes in narratives, identifying verbs, phrases or idioms assumed to be the textual markers of emotional expressions, and temporal perspectives [21,22]. The Spatio-Temporal Perspective Module encompasses the forms of Retrospective and Re-experiencing perspectives [13], which we used in this study to group participants. Subsequently, we examined the differences between the Retrospective and Re-experiencing groups’ narratives in terms of the use of words and expressions identified by the Psychological Perspective module (as presented in Figure 1). NarrCat defines Psychological Perspective as the range of mental states attributed to individuals and groups in texts, aiding in the exploration of mental processes, thoughts, and beliefs. It consists of the Emotion, Cognition, and Intentionality-Agency submodules. The Emotional submodule categorizes emotions into Positive (e.g., “joy”, “contentedness”, “hope”) and Negative (e.g., “suffering”, “disappointment”, “uneasiness”) groups, while the Cognition submodule includes mental verbs (“generalize”, “ponder”), nouns (“thought”, “decision”, “idea”), and mental idioms denoting cognitive processes (Laszlo et al., 2013 [21]). Intentionality can be expressed by intentional auxiliary verbs (such as “want”, “will”, and “wish”) and by intentional nouns (“goal”, “plan”), adverbs (“goal-mindedly”), adjectives (“intentional”), and postpositions. The more intentional words and expressions are used by the narrator, the more goal-oriented and efficient the agent appears to be [21,22].

#### 2.4.2. Analytic Processes

Statistical analyses were completed using the Jamovi statistical platform [23]. To explore the relationship between time perspectives and the variables under study, we conducted a cluster analysis. We used hierarchical clustering (Ward’s method and squared Euclidean distances) to determine the number of potential clusters. This is an agglomerative method that clusters similar elements, minimizing the variance within clusters at each stage of clustering. As a second step, K-means cluster analysis was conducted, with the number of clusters confirmed by the hierarchical clustering. K-means analysis ensures high intra-class similarity and low inter-class similarity and separates observations into uniform groups. Following the cluster analysis, a univariate analysis of variance was conducted to examine the differences in time perspectives between the clusters.

The narrative variables of the high-point and low-point episodes were examined using a non-parametric Mann–Whitney U test, suitable for non-normally distributed data. In the data analysis, we compared the frequencies of the narrative markers on emotions and psychological perspective of the high- and low-point episodes of the time perspective clusters and compared their well-being and subjective evaluation of physical impairment.

Distributions of patients’ demographic and clinical characteristics were compared for time perspective clusters using Chi-squared tests.

For all statistical analyses, a *p*-value of <0.05 was considered significant.

## 3. Results

### 3.1. Characterization of the Clusters

For the time perspective, hierarchical cluster analysis indicated a two-cluster layout. Subsequently, K-means cluster analysis was conducted, with two clusters for the relative frequency values of temporal perspectives in high-point episodes. Cluster 1—labeled as Retrospective (N = 25; mean of Retrospective perspective: 0.62, SD: 0.09; mean of Re-experiencing perspective: 0.31; SD: 0.08)—consisted of individuals who had a higher relative frequency of Retrospective perspective expressions and lower Re-experiencing expressions. Cluster 2—labeled as Re-experiencing (N = 35; mean of Re-experiencing perspective: 0.56, SD:0.08; mean of Retrospective perspective: 0.35; SD: 0.08)—consisted of individuals who had a higher relative frequency of Re-experiencing expressions and lower Retrospective expressions (Appendix A). According to the univariate analysis of variance, the difference between clusters was significant in the Retrospective perspective (F(1.58) = 149.21; *p* < 0.001) and the Re-experiencing perspective (F(1.58) = 138.92; *p* < 0.001). By examining the demographic characteristics of the two groups, the Chi-squared tests indicated that there was no statistical difference between the two groups in the distribution of demographic and clinical characteristics (as summarized in Table 2).

### 3.2. Association between the Temporal Perspective Adopted during High-Point Episodes and the Narrative Characteristics of Low-Point Episodes

**Figure 2:**
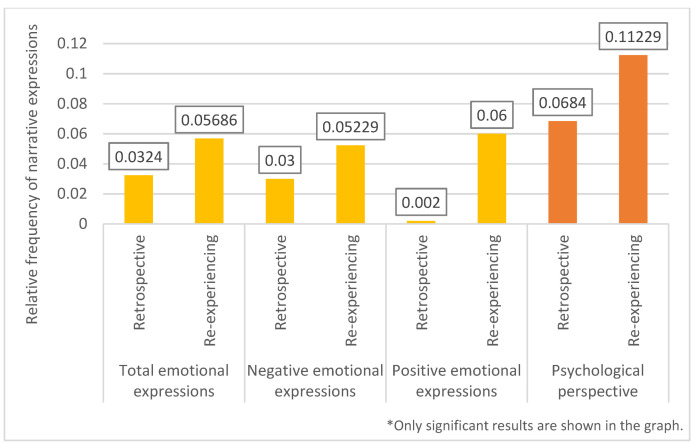
Narrative frequencies of emotional expressions and psychological perspective in the two cluster’s low-point episodes.

Subsequently, intergroup differences in psychological state, physical functioning, and narrative variables between these two groups were investigated using the Mann–Whitney U test. In low-point episodes, the Re-experiencing group used more psychological perspectives (U(58) = 223; *p* < 0.01). Regarding the Psychological Perspective submodules, the Re-experiencing group showed heightened emotional frequency (Mann–Whitney U Test; total: U(58) = 280; *p* < 0.05; positive: U (58) = 328; *p* < 0.05; negative: U(58) = 278; *p* < 0.05) in their low point episodes (Figure 2). In terms of Cognition (U(58) = 331; *p* > 0.05) and Intention (U(58) = 337; *p* = 0.09) submodules, the two groups did not differ significantly. However, no significant difference emerged in the Emotions (Mann–Whitney U Test; total: U (58) = 377, *p* > 0.05; positive: U (58) = 352, *p* > 0.05; negative: U (58) = 433, *p* > 0.05), Psychological Perspective (U (58)= 0.424, *p* > 0.05), Cognition (U (58) = 429, *p* > 0.05) and Intention (U (58) = 416, *p* > 0.05) features of the Retrospective and Re-experiencing groups regarding the high-point episodes.

### 3.3. Link between Temporal Perspectives and Self-Reported Mental Well-Being and Physical Impairment

Based on the above-mentioned global cut-off scores of the HADS questionnaire, the mean values within our study group were situated at subclinical levels for both dimensions (anxiety mean: 7.69. SD: 4.23; depression mean: 6.22; SD: 4.16). Regarding physical functioning, our sample was generally characterized by mild physical limitations (HAQ mean: 0.86, SD: 0.69). Based on HAQ global cut-off scores, 60% (15 people) of the Retrospective group had mild physical symptoms and 40% (10 people) had moderate symptoms. In the Re-experiencing group, 60% (21 people) had mild impairment, 40% (14 people) had moderate physical limitations, and no participant reached the level of severe disability.

Using the cut-off scores of the HADS questionnaire, 72% (18 people) of the Retrospective group showed no clinically relevant anxiety symptoms, 8% (2 people) had moderate anxiety symptoms, and 20% (5 people) were likely to have a clinical level of anxiety. In the Re-experiencing group, 69% (24 people) showed no clinically relevant anxiety symptoms, 14% (5 people) have moderate anxiety symptoms, and 17% (6 people) had probably clinically significant anxiety symptoms. In terms of depressive symptoms, 72% (18 people) of the Retrospective group had no clinically relevant depressive symptoms, 16% (4 people) had moderate depressive symptoms and 12% (3 people) were likely to have clinical depression. Moreover, 71% (25 people) of the Re-experiencing group had no clinically relevant depressive symptoms, 13% (4 people) had moderate symptoms and 17% (6 people) were likely to have clinical depression.

## 4. Discussion

The Psychological Perspective module encompasses emotional and cognitive dimensions, with the Re-experiencing group demonstrating a significantly higher frequency of emotional expressions compared to the Retrospective group. In a recent study, Polya et al. [24] discovered that in product reviews, a higher proportion of Retrospective perspectives was associated with lower emotional involvement, while a higher proportion of the present-tense perspective indicated higher emotional involvement. Consistent with these findings, our results suggest that a more intense recall style is linked to more vivid emotional content in low-point episodes. The considerably elevated the occurrence of emotions within the low-point episodes of the Re-experiencing group may signify distinct patterns in memory processing, elaboration, and adaptive responses. Notably, our analysis revealed differences primarily in the Emotional submodule, indicating that high-point events in RA patients are organized similarly in terms of emotional content, differing only in temporal perspective. The vivid and present tense narrative style is closely related to the concept of Mental Time Travel (as described in Quoidbach et al. [8]—hence, the Re-experiencing group presumably uses this savoring technique, while the other group adopts a Retrospective approach. Research indicates that savoring plays a crucial role in mental health, and an increased intensity and frequency of positive emotions serves as a mediator between resilience and psychological well-being in the general population [8,9]. Enhanced savoring abilities may broaden cognitive and behavioral repertoires, bolstering resilience and subsequently improving mental health outcomes. Savoring-based intervention reduced pain intensity, increased positive emotions, and reduced anhedonic symptoms in RA patients [6]. Moreover, savoring has been linked to improved happiness and life satisfaction in cancer patients, with high savoring levels associated with reduced depressive symptoms independent of physical symptoms caused by the disease [10,11]. The broaden-and-build model [9] suggests that positive emotions enhance adaptability and cognitive flexibility by broadening thought–action tendencies. However, our study did not find evidence that positive emotions are associated with increased cognitive functioning. There was no significant difference between the two groups in the Cognition and Intention submodules, which may be due to the effect of emotional responses to emotionally intense life experiences elicited by recollecting these memories.

Surprisingly, no significant difference emerged between the Retrospective and Re-experiencing groups regarding any other narrative characteristics of high-point episodes—such as Emotion, Cognition or Psychological Perspective. One potential explanation for this outcome could be the uniformity of emotional experiences associated with positive life events: people tend to feel happiness in similar ways, but suffering can vary greatly. Berntsen et al. [25] obtained similar results in their study, which investigated the correlation between the most significant negative and positive life events and well-being among approximately 2000 adults in their sixties. Their findings indicate that positive and negative autobiographical events relate differently to life-story narratives and well-being: positive events often become central through their correspondence with cultural norms, thus being less unique, while negative events tend to become central due to mechanisms linked to emotional distress. This difference may explain the emotional richness observed in low-point episodes and the more uniform organization of high-point episodes in our own results. Additionally, research on narrative life stories suggests that positive events are more easily processed and integrated into personal narratives, potentially resulting in less emotional variance, which might explain why there is no difference in emotions between the two groups for the high points [3]. The Re-experiencing strategy might show the emotional work of psychological processing and how it appears in negative stories.

This raised the question of whether the Re-experiencing strategy in recalling positive life events is an adaptive approach, as indicated by Quoidbach et al.’s [8] research, so we examined whether there were differences in mental and physical well-being between the Re-experiencing and Retrospective groups. There are no significant differences between groups in terms of psychological state or physical impairment. Although our assumption was well-founded, since several previous studies have found a link between the more intense recollection and reliving of positive experiences (savoring strategies) and well-being variables [8,10,11], the present results cannot confirm the adaptive nature of the Re-experiencing strategy.

An explanation for the lack of difference between the two groups’ mental health and physical state variables and narrative traits could stem from the prevalent symptom-oriented approach in medicine. Through negatively worded items, patient questionnaires often emphasize negative aspects of well-being, directing attention to pain and limitations [12]. In summary, although the two temporal perspective groups may not differ in symptoms, they may differ in positive functioning. Therefore, it is important to capture these positive aspects using questionnaires that focus on bolstering health and well-being. Further studies are needed to answer this question and to explore narrative and language variable change during autoimmune disease interventions, as well as to determine the potential for guided memory recall in RA patients as a basis for psychosocial interventions.

### 4.1. Limitations, Future Directions

The results suggest avenues for future research, emphasizing the relationship between temporal perspectives and well-being. The study’s small sample sizes, gender imbalance and limited geographic representation indicate the need for broader population inclusion in qualitative research for a more comprehensive investigation. Furthermore, we must acknowledge that the results are derived from a single clinical study, which included only hospitalized patients undergoing treatment. Consequently, the study has several inherent potential biases beyond geographical limitations and the small sample size, so it is crucial to address these biases on the generalizability of the findings to patients who are not undergoing hospital treatment. It is also important to note that the sample was in good mental and physical condition (as indicated by their willingness to participate in the research) and had been living with the disease for a longer period of time (>10 years). Additionally, the well-known limitations of questionnaire research may have contributed to the results obtained. Yet we still believe that future investigations could benefit from eliciting patients’ positive emotions and enjoyments, potentially revealing subgroup distinctions in responses to peak experiences.

As mentioned in the limitations, the relatively good condition of the patients implies that the sample is not representative of all RA patients across the full spectrum of symptom severity. Therefore, the authors aim to include patients with more severe conditions to cover a broader spectrum of mental and physical states. Future research would benefit from using more nuanced approaches on a broader sample, including newly diagnosed patients and exploring their disease-related narratives. The longitudinal implementation of the research may further elucidate the association between temporal perspectives and well-being in RA patients.

### 4.2. Clinical Implications

Positive emotions are a promising target for interventions in chronic pain. Related to novel research approaches, investigating patient’s enjoyment can function as a positive intervention, generating clinical value by facilitating discussions about positive life aspects. Constructing interventions based on the recall of positive experiences can boost motivation, enhance enjoyment, and positively influence behaviors linked to seeking pleasure [12]. The outcomes of this positive emotion-enhancing intervention, known as Savoring Meditation, conducted with RA patients, demonstrate the effectiveness of these techniques in pain self-management [6]. A longitudinal positive-emotion induction intervention on RA-associated pain and disability also shows encouraging results: intervention consists of recalling happy memories twice a day, and a few weeks after the intervention, there was found to be a positive shift in depression, anxiety, and physical impairment [17].

## 5. Conclusions

The present research has contributed to a better understanding of RA patients’ life experiences and the role of different emotions in their memory recall strategies. Our results may enhance RA practitioners’ insights into resources influencing illness adaptation and support them in guiding client-focused methods. The main result was that individuals who vividly recall positive life events delve deeper when it comes to negative memories. Mental Time Travel into the high-point episodes might be a marker of a psychological resource that supports narrators’ more intense emotional elaboration of stressful memories. Moreover, the Re-experiencing group’s heightened positive emotion frequency during low-point episodes may stem from emotion regulation triggered by negative memories; however, it is still under question whether this strategy favors mental well-being and better functioning.

## Figures and Tables

**Table 1:** Two forms of narrative perspective.

Narrative Perspective	Narrator’s Location	Life-Story Content Location
Retrospective	present	past
Re-experiencing	present	present

**Table 2:** Demographic characteristics of the sample.

Demographic Categories	Total (*n* = 60)	Group 1 Retrospective (*n* = 25)	Group 2 Re-Experiencing (*n* = 35)	*p*-Value
Gender				
Male	9 (15%)	3 (12%)	6 (17%)	*p* = 0.582
Female	51 (85%)	22 (88%)	29 (83%)	
Age				
35–49	14 (23%)	6 (24%)	8 (23%)	*p* = 0.223
50–64	19 (32%)	5 (20%)	14 (40%)	
65–80	27 (45%)	14 (56%)	13 (37%)	
Marital status				
Married	41 (68%)	16 (64%)	25 (83%)	*p* = 0.542
Divorced/widowed	19 (32%)	9 (36%)	10 (17%)	
Employment				
Full time/part time	16 (26%)	6 (24%)	10 (29%)	*p* = 0.693
Retired/disability pension	44 (74%)	19 (76%)	25 (71%)	
Highest level of education				
Higher education/high school	39 (35%)	16 (64%)	23 (66%)	*p* = 0.891
Elementary/undergraduate	21 (65%)	9 (36%)	12 (34%)	
Disease duration				
10 years >	5 (8%)	3 (12%)	2 (6%)	*p* = 0.385
10 years <	55 (92%)	22 (88%)	33 (94%)	

**Figure A1:**
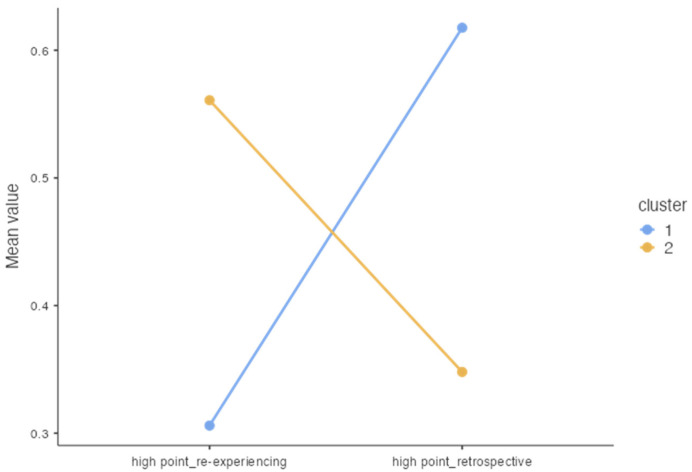
Plot of the means based on the means of relative frequencies of Retrospective and Re-experiencing perspectives across groups.

## Data Availability

The sensitive life story data is protected due to ethical considerations, and because all interviews were conducted in Hungarian, and transcriptions were also in Hungarian.
